# Development of a Novel Disturbance Observer Based Fractional Order PD Controller for a Gun Control System

**DOI:** 10.1155/2014/284636

**Published:** 2014-01-28

**Authors:** Qiang Gao, Liang Zheng, Jilin Chen, Li Wang, Yuanlong Hou

**Affiliations:** ^1^School of Mechanical Engineering, Nanjing University of Science and Technology, Nanjing 210014, China; ^2^Military Representative Office of the General Armaments Department in Wuhan Area, Wuhan 430073, China

## Abstract

Motion control of gun barrels is an ongoing topic for the development of gun control equipment (GCE) with excellent performances. In this paper, a novel disturbance observer (DOB) based fractional order PD (FOPD) control strategy is proposed for the GCE. By adopting the DOB, the control system behaves as if it were the nominal closed-loop system in the absence of disturbances and uncertainties. The optimal control parameters of the FOPD are determined from the loop-shaping perspective, and the *Q*-filter of the DOB is deliberately designed with consideration of system robustness. The linear frame of the proposed control system will enable the analysis process more convenient. The disturbance rejection properties and the tracking performances of the control system are investigated by both numerical and experimental tests, the results demonstrate that the proposed DOB based FOPD control system is of more robustness, and it is much more suitable for the gun control system with strong nonlinearity and disturbance.

## 1. Introduction

Gun control equipment (GCE) has been extensively regarded to be one of the key components of fire control systems (FCSs). Generally, the motion robustness and the motion accuracy of the gun barrel are definitely the two main challenges associated with the developments of the GCE with excellent performances [1, 2]. The motion control of gun barrels is an ongoing topic due to certain extremely complicated segments with strong nonlinearities and uncertainties, such as the time-varying parameters induced by the varying working conditions, the externally applied random loads, and the complex friction forces between the cannon and the trunnion [3–5]. To eliminate these nonlinearities induced negative effects, a dominant method is the application of the well-known proportional-integral-derivative (PID) based feedback control strategy [3, 4, 6]. However, due to the inherent nonlinearities as mentioned above, it is hard for the linear PID control strategy to achieve excellent control performances, and the unsuitable PID controller will significantly limit the dynamic performances of the GCE. Devoted to compensating for the nonlinearities and achieving high robustness, considerable work has been done by means of employing certain adaptive control strategies, such as fuzzy control, adaptive sliding mode robust control (SMC), adaptive equivalent disturbance compensation control, and active disturbance rejection control (ADRC) [2, 7–10]. All these complex nonlinear strategies based methods can well improve the tolerance of the system to various uncertainties and disturbances, but only at expense of the positioning accuracy and the response rate.

Fractional order PID (FOPID) type controllers, which were proposed by Podlubny, are extended versions of conventional integer order PID controllers [11]. When comparing with conventional PID controllers, this sort of controllers possess unique characteristics including abundant dynamics, high robustness, and fine tracking accuracy [12–14]. Practical comparisons suggested that the FOPID control system had much better control performances than the ADRC, the optimal PID, and the SMC controllers [15]. Although the FOPID controllers are much less sensitive to external disturbances, the tracking accuracy will also be deteriorated when facing much larger disturbances and much stronger nonlinearities. Besides, the model uncertainties of the plant will lead to deviations of the optimal control parameters which may be determined by certain state-of-the-art tuning methods [16–19], resulting in suboptimal control performances.

Motivated by this, a novel disturbance observer (DOB) based fractional order PD (FOPD) controller is proposed to further achieve high precision control in the presence of various disturbances and plant uncertainties. The DOB is employed here due to its simple structure as well as a powerful ability to reject disturbances and compensate for plant uncertainties [20–22]. In addition, the linearity of the FOPD and the DOB will provide much more convenience to analyze and tune the control systems. The remainder of this paper is summarized as follows: in Section 2, the nominal physics model of the gun control system is developed; the basic principle of the DOB based FOPD control system is introduced, and the optimal parameter determination procedure is then introduced in Section 3; numerical simulations and experiment tests of the control system are detailed in Sections 4 and 5, respectively; finally, the main conclusions of this paper are drawn in Section 6.

## 2. Modeling of the AC Servo System of the GCE

**Figure 1:**
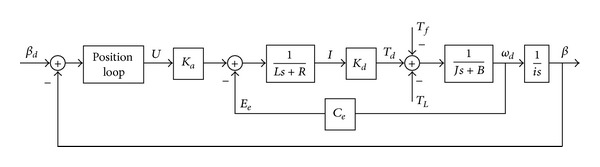
Schematic of the AC servo system of GCEs.

The schematic of the AC servo system utilized in the GCE is illustrated in Figure 1, where *β*_*d*_ and *β* represent the desired angle position and the real angle position of the cannon, respectively. *U* is the control voltage; *K*_*a*_ is the amplify gain; *K*_*d*_ is the motor torque factor. *T*_*d*_, *T*_*L*_, and *T*_*f*_ are the motor torque, load torque disturbance, and friction torque disturbance, respectively. *R* and *L* represent the resistance and inductance of the motor armature circuit, respectively. *E*_*e*_ is the counter-electromotive force (CEMF) of the motor armature and *C*_*e*_ denotes the CEMF coefficient. *J* is the total moment of inertia to the rotor; *B* is the viscous friction coefficient; *ω*_*d*_ is the angular velocity of the motor; *i* is the moderating ratio; *s* denotes the Laplace operator.

Generally, the current time constant is much smaller than the mechanical time constant; the delay of the current response can be neglected and it yields

(1) $$\begin{array}{c} \frac{1}{Ls+R}=\frac{1}{R}\frac{1}{Ls/R+1}\approx \frac{1}{R}. \end{array}$$

The motor torque *T*_*d*_ is given as follows:

(2) $$\begin{array}{c} T_{d}=-\frac{K_{d}C_{e}}{R}\omega_{d}+\frac{K_{d}K_{a}\;}{R}U. \end{array}$$

According to the equilibrium equation of the torques, we can obtain

(3) $$\begin{array}{c} T_{d}-T_{L}-T_{f}=Ji\ddot{\beta }+Bi\dot{\beta }. \end{array}$$

Substituting (2) into (3) yields

(4) $$\begin{array}{c} Ji\ddot{\beta }+Bi\dot{\beta }=-\frac{K_{d}C_{e}}{R}\omega_{d}+\frac{K_{d}K_{a}}{R}U-T_{L}-T_{f}. \end{array}$$

When the motor torque and load torque disturbance are ignored, the govern principle of the AC servo system can be obtained:

(5) β¨+(BJ+KdCeJR)β˙=KdK  a  iJRU.

The transfer function of the AC servo system could be obtained by taking Laplace transformation of (4), which could be obtained by

(6) $$\begin{array}{c} P\left(s\right)=\frac{\beta \left(s\right)}{U\left(s\right)}=\frac{K_{d}K_{a}\;}{i}\frac{1}{s\left(JRs+BR+K_{d}C_{e}\right)}. \end{array}$$

Thus, the phase and the gain of the plant can be given by

(7) $$\begin{array}{c} P\left(j\omega \right)=\frac{K_{d}K_{a}}{iJR}\frac{JR}{{\left(j\omega \right)}^{2}JR+\left(BR+K_{d}C_{e}\right)\left(j\omega \right)},\\ \left|P\left(j\omega \right)\right|=\frac{K_{d}K_{a}}{i\omega }\frac{1}{\sqrt{\omega^{2}{\left(JR\right)}^{2}-\left(BR+K_{d}C_{e}\right)}},\\ \text{Arg}\left[P\left(j\omega \right)\right]=-\frac{\pi }{2}+\arctan\frac{\left(BR+K_{d}C_{e}\right)}{JR\omega }. \end{array}$$

## 3. Development of the Novel Control Strategy

### 3.1. A Preliminary to the FOPD Controller

According to the works of Podlubny, the FOPD controller is introduced. The control law of such a controller can be written as [11]

(8) $$\begin{array}{c} u\left(t\right)=k_{p}e\left(t\right)+k_{d}D_{t}^{\lambda }e\left(t\right), \end{array}$$

where *k*_*p*_ and *k*_*d*_ are proportion and differentiator gain, respectively. *D*_*t*_^*λ*^ *f*(*t*) = _*t*_0__^^ *D*_*t*_^*λ*^ *f*(*t*) is the noninteger order fundamental operator; *λ* denotes the order of the differentiator.

By taking Laplace transformation of (8), the transfer function of the FOPD can be obtained by

(9) $$\begin{array}{c} C\left(s\right)=k_{p}+k_{d}s_{}^{\lambda },\\ C\left(j\omega \right)=k_{p}+k_{d}{\left(j\omega \right)}_{}^{\lambda }. \end{array}$$

Since *j* ^*α*^ = (*e* ^*j*(*π*/2)^)^*α*^ = cos⁡⁡(*απ*/2) + *j*sin(*απ*/2), the transfer function of FOPID could be rewritten as

(10) $$\begin{array}{c} C\left(j\omega \right)=\left[k_{p}+k_{d}\omega^{\lambda }\cos\left(\frac{\pi }{2}\lambda \right)\right]+jk_{d}\omega^{\lambda }\sin\left(\frac{\pi }{2}\lambda \right). \end{array}$$

The phase and the gain of the FOPD could be further given as

(11) $$\begin{array}{c} \left|C\left(j\omega \right)\right|=\sqrt{{\left[k_{p}+k_{d}\omega^{\lambda }\cos\left(\frac{\pi }{2}\lambda \right)\right]}^{2}+{\left[k_{d}\omega^{\lambda }\sin\left(\frac{\pi }{2}\lambda \right)\right]}^{2}},\\ \text{Arg}\left[C\left(j\omega \right)\right]=\arctan\frac{k_{d}\omega^{\lambda }\sin\left(\left(\pi /2\right)\lambda \right)}{k_{p}+k_{d}\omega^{\lambda }\cos\left(\left(\pi /2\right)\lambda \right)}. \end{array}$$

### 3.2. The DOB Based FOPD Control Strategy

**Figure 2:**
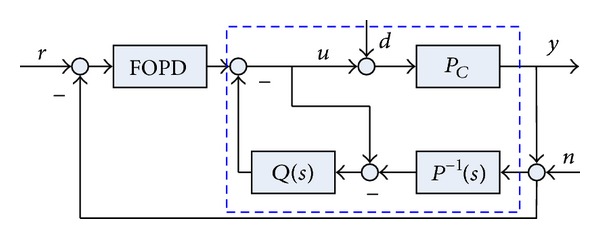
Schematic of the DOB based FOPD control system.

Schematic of the DOB based FOPD control system is illustrated in Figure 2, where *P*_*C*_ and *P*(*s*) represent the single-input single-output real plant and its nominal model is shown in (6), respectively. The dashed block represents the actual plant *P*_*C*_ augmented with the DOB. Generally, the DOB and the FOPD can be referred to as an inner-loop controller and an outer-loop controller, respectively. *Q*(*s*), also known as the *Q*-filter, is a stable low-pass filter with the unity dc gain. The signals *d* and *n* represent the input disturbance and the system noise with high frequency, respectively. From the control block diagram shown in Figure 2, the output of the system can be obtained by [20]

(12) $$\begin{array}{c} y\left(s\right)=T_{yr}\left(s\right)r\left(s\right)+T_{yd}\left(s\right)d\left(s\right)-T_{yn}\left(s\right)n\left(s\right), \end{array}$$

where

(13) $$\begin{array}{c} T_{yr}=\frac{P_{C}PC}{P\left(1+P_{C}C\right)+Q\left(P_{C}-P\right)},\\ T_{yd}=\frac{P_{C}P\left(1-Q\right)}{P\left(1+P_{C}C\right)+Q\left(P_{C}-P\right)},\\ T_{yn}=\frac{P_{C}\left(PC+Q\right)}{P\left(1+P_{C}C\right)+Q\left(P_{C}-P\right)}. \end{array}$$

In the low frequency range for which *Q*(*jω*) ≈ 1, it follows that

(14) $$\begin{array}{c} T_{yr}\approx \frac{PC}{1+PC},\;\;T_{yd}\approx 0,\;\;n\left(j\omega \right)\approx 0. \end{array}$$

Thus, the output of the plant can be reduced to [20]

(15) $$\begin{array}{c} y\left(s\right)=\frac{P\left(s\right)C\left(s\right)}{1+P\left(s\right)C\left(s\right)}r\left(s\right). \end{array}$$

From the structure of the equivalent transfer function of the closed-loop system, it implies that the real uncertain closed-loop system with the DOB behaves as if it were the nominal closed-loop system in the absence of disturbance. On the other hand, the DOB is used as a part of controller compensating for disturbances. Also, the DOB has the property of model shaping such that it forces the input-output behavior of real plant to follow that of nominal plant [20, 22].

### 3.3. The Optimal Control Parameter Determination

As is aforementioned, the control system consists of the outer-loop and the inner-loop controllers. As for the outer-loop controller, namely, the FOPD, *k*_*p*_, *k*_*d*_, and *λ* are the three main parameters that determine the performances of the control system. Here, three specifications to be met are applied [10, 16–19] from the loop-shaping perspective: namely, the phase margin specification, the robustness to gain variations, and the gain crossover frequency specification, which will be detailed below.

(a) Phase margin specification:
  (16) $$\begin{array}{c} \text{Arg}\left[P\left(j\omega_{c}\right)C\left(j\omega_{c}\right)\right]=-\pi +\phi_{m}. \end{array}$$

That is,

(17) arctankdωcλsin((π/2)λ)kp+kdωcλcos⁡((π/2)λ)  +arctan(BR+KdCe)JRωc=−π2+ϕm,

where *ω*_*c*_ is the gain crossover frequency interested and *ϕ*_*m*_ is the phase margin required.

(b) Robustness to gain variations:
  (18) $$\begin{array}{c} {\frac{d\text{Arg}\left[P\left(j\omega \right)C\left(j\omega \right)\right]}{d\omega }|}_{\omega =\omega_{c}}=0. \end{array}$$

With this condition, the phase Bode plot is flat at the gain crossover frequency. It means that the system is more robust to gain changes and the overshoots of the response are almost the same.

(c) Gain crossover frequency specification: at the gain crossover frequency point, the amplitude of the open-loop transfer function should be zero:
  (19) $$\begin{array}{c} {\left|G(j\omega_{c})\right||}_{\text{dB}}={\left|P(j\omega_{c})C(j\omega_{c})\right||}_{\text{dB}}=0. \end{array}$$

That is,

(20) KdKaiωc ×[kp+kdωcλcos⁡((π/2)λ)]2+[kdωcλsin((π/2)λ)]2ωc2(JR)2−(BR+KdCe)=1.

As for the inner control system, the *Q*-filter may play an important role in the robustness and the disturbance rejection performance. Generally, there are three important factors in designing a *Q*-filter, namely, the filter time constant, the numerator order, and the denominator order. Hereby, the filter of the following form is employed [22]:

(21) $$\begin{array}{c} Q_{m,n}\left(s\right)=\frac{\sum_{i=1}^{n}a_{m,i}{\left(\tau s\right)}^{i}}{{\left(\tau s+1\right)}^{m}}, \end{array}$$

where *τ* is the filter time constant, *a*_*m*,*i*_ is the binomial coefficient, and *m* and *n* are the numerator and the denominator orders, respectively. Choi et al. [22] suggested that (1) the smaller the relative degree, the better the robustness; (2) the larger the denominator order, the better the robustness. Thus, with additional consideration of computation efficiency, *m* and *n* are chosen to be 3 and 1, respectively. Since the filter time has no obvious relationship with the robustness, it is chosen to be 0.02.

## 4. Numerical Simulation

As for the description of the AC servo system, the system parameters are chosen as follows: *J* = 0.0352 kg·m^2^, *K*_*d*_ = 0.195 N·m/A, *C*_*e*_ = 0.195 V/(rad·s^−1^), *R* = 0.07 Ω, and *B* = 0.000143 V/(rad·s^−1^). As for the design specifications of the controller, the interested crossover frequency is set as 5 Hz with respect to practical motions of the gun control servo system, and the required phase margin is set as *ϕ*_*m*_ = *π*/4. The parameters of the FOPD are solved according to (17)–(20) by means of the nonlinear solving module of the software MATLAB. The determined controller parameters are set as *k*_*p*_ = 1.2, *k*_*d*_ = 0.16, and *λ* = 0.26.

**Figure 3:**
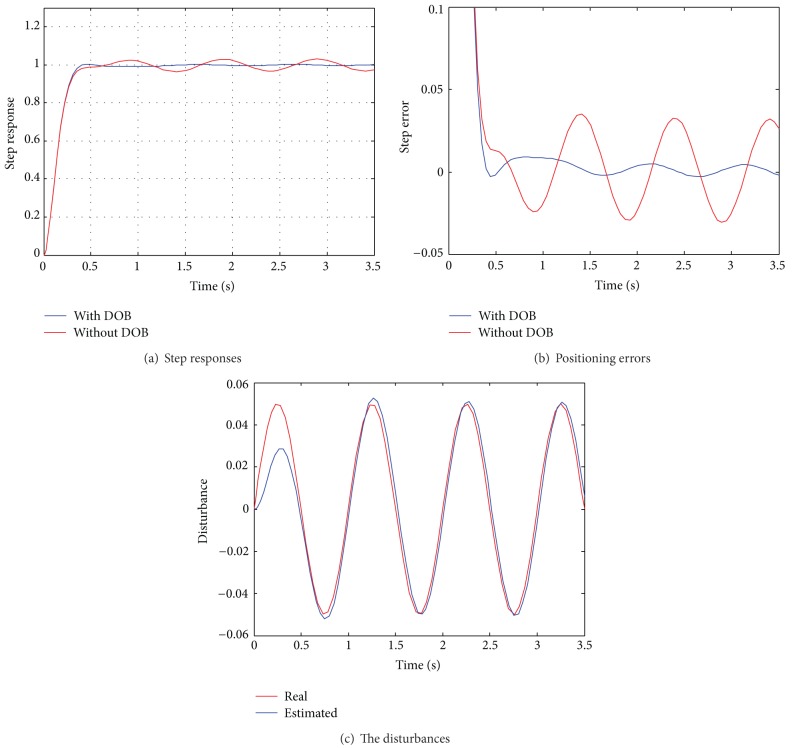
Step responses of the control system.

To investigate the disturbance rejection capacity of the proposed DOB based FOPD control strategy, step response with sinusoid disturbance is investigated where the frequency and amplitude of the predefined disturbance are 1 Hz and 0.05, respectively. The obtained response results are illustrated in Figure 3(a), and the obtained positioning errors are presented in Figure 3(b). To examine the efficiency of the DOB, the estimated and the employed disturbances are shown in Figure 3(c).

As shown in Figure 3(a), the step responses of the system with and without DOB have the same rising features, the arise time is about 0.18 s, and no overshoots can be observed. The results demonstrate the efficiency of the developed FOPD controller for the nominal control system. From the positioning errors shown in Figure 3(b), the positioning error of the system without DOB is about ±0.034, while that of the system with DOB is about ±0.0015, which is about twentieth of that of the system without DOB. The results indicate that the disturbance rejection capacity has been significantly improved, attributing to the good estimation capacity of the DOB, which can also be verified by the estimation results shown in Figure 3(c). As shown in Figure 3(c), a good satisfaction between the estimated and the practical disturbances is achieved with certain delay. In addition, the exponential convergence at the beginning phase can be observed, which is a notable advantage of enhancing system stability in practice. Overall, the simulation results well verify the efficiency of the proposed DOB based FOPD control strategy.

## 5. Experiment Results and Discussion

### 5.1. The Experiment Setup

**Figure 4:**
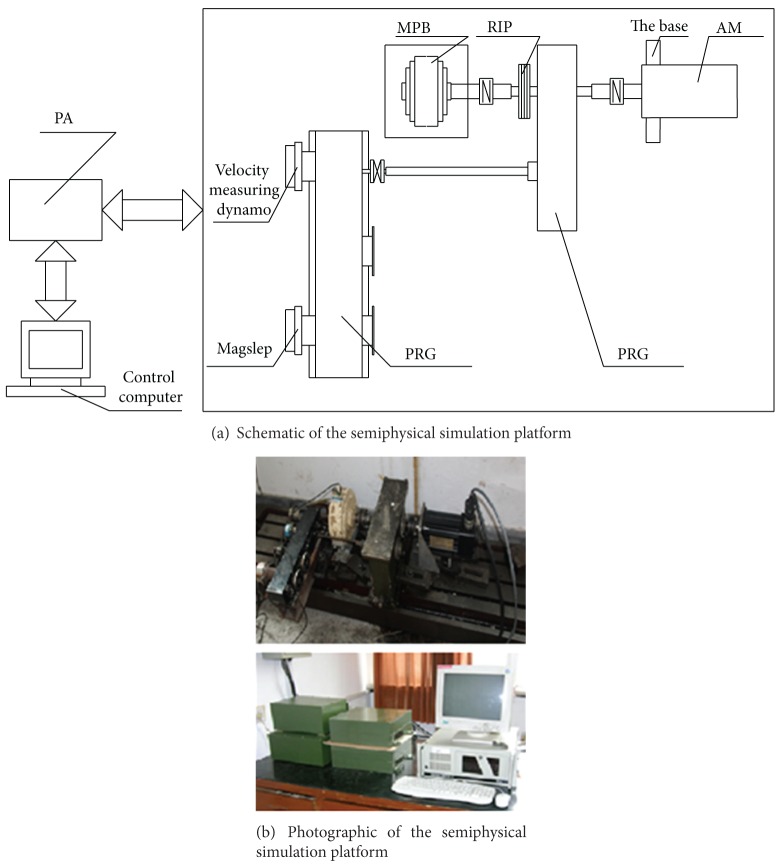
Semiphysical simulation platform.

To investigate the efficiency of the proposed control strategy applying for the GCE, a semiphysical simulation platform is constructed to simulate the practical working conditions of the gun control system. The structure diagram and the photographic diagram of the platform are, respectively, illustrated in Figures 4(a) and 4(b). From the components as shown in Figure 4, the platform mainly consists of seven parts, namely, the control computer, the sensor system for measurement, the power amplifier (PA), the precision reduction gearbox (PRG), the loading fixture (LF), the actuating motor (AM), and the test bed. The loading fixture consisting of the rotational inertia plate (RIP) and the magnetic powder brake (MPB) is employed here for the simulation of the rotational inertia, the load torque, and the frictional resistance moment, which would be generated under the real working conditions. The rotational inertia variations of the loads can be well simulated by changing the RIP, and, similarly, the variations of the load torque and the frictional resistance moment can also be well simulated by controlling the output torque of the MPB.

### 5.2. Control Performances of the Gun Control System

To estimate tracking performances of the gun control system, constant speed tracking experiments are conducted on the semiphysical simulation platform. To investigate the robustness of the system to external disturbances, the equivalent disturbance for simulating external load variations and friction forces is employed by giving control signals to the MPB to generate the frictional resistance moment, which is about *T*_*f*_ = 6 + 4sin*πt* (N·m).

**Figure 5:**
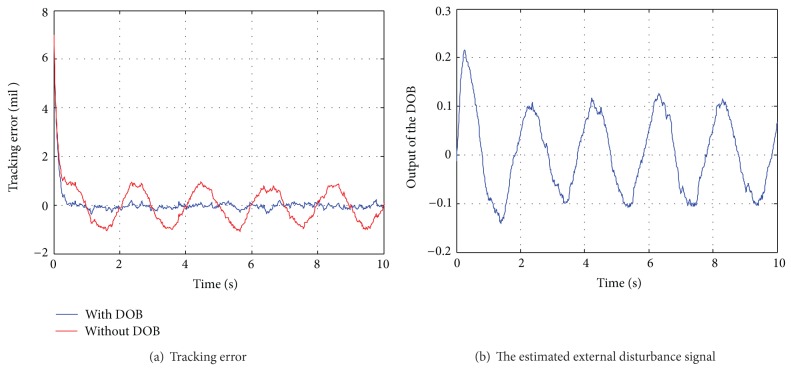
Tracking performance of the control system with external disturbance.

To avoid repetition, Figure 5(a) only illustrates the tracking error of the control system with constant angular speed ${\dot{y}}_{d}=1^{{}^{\circ}}/\text{s}$; the estimated external disturbance signal is illustrated in Figure 5(b). As shown in Figure 5(a), the response time of the two control systems is about 0.26 s. The tracking error of the DOB based FOPD control system in the steady state is about 0.5 mil which is about 26.32% of that of the control system without the DOB. From the estimated disturbance signal shown in Figure 5(b), it verifies that the DOB can effectively estimate the disturbances. The enhanced tracking accuracy demonstrates that the DOB based FOPD control strategy can efficiently improve the robustness of the control system, and it indeed outperforms the conventional FOPD control strategy.

## 6. Conclusions

In this paper, a novel disturbance observer (DOB) based fractional order PD control strategy is proposed for the gun control equipment. By adopting the DOB, the control system behaves as if it were the nominal closed-loop system in the absence of disturbances and uncertainties. The optimal control parameters of the FOPD for the nominal plant are determined from the loop-shaping perspective, and the *Q*-filter of the DOB is deliberately designed with consideration of system robustness. Numerical simulation of step response of the system with external disturbance demonstrates that the rise time of the control system with and without the DOB can reach up to 0.18 s, and there are no undesired overshoots. In addition, the positioning error of the system with the DOB is about twentieth of that of the system without DOB, well demonstrating the disturbance rejection capacity of the DOB. By experimentally conducting the constant speed tracking, the tracking error of the system with the DOB is about 0.5 mil which is about 26.32% of that of the system without the DOB. All the results demonstrate that the proposed DOB based FOPD control strategy can efficiently reject external disturbances and system uncertainties, and it is of much superior control performances than the FOPD control strategy.

## References

[1] Kumar G, Tiwari PY, Marcopoli V, Kothare MV A study of a gun-turret assembly in an armored tank using model predictive control.

[2] Gao Q, Sun Z, Yang G, Hou R, Wang L, Hou Y (2012). A novel active disturbance rejection-based control strategy for a gun control system. *Journal of Mechanical Science and Technology*.

[3] Hao X, Wei Y, Wendong Z, Xiaomin D, Haiyang Y The application of dual-PID regulation based on sliding mode control in a tank artillery stabilizer.

[4] Shen LJ, Bao Y, Cai JP (2011). Adaptive control of uncertain gun control system of tank. *Applied Mechanics and Materials*.

[5] Feng L, Ma X, Yan Z, Li H (2007). Method of adaptive fuzzy sliding mode control of gun control system of tank. *Electric Machines and Control*.

[6] Xia Y, Dai L, Fu M, Li C, Wang C (2013). Application of active disturbance rejection control in tank gun control system. *Journal of the Franklin Institute*.

[7] Ma XJ, Feng L, Yuan D (2010). Overview of adaptive compensation control of nonlinearity in tank gun control system. *Fire Control and Command Control*.

[8] Liang F, Xiao-jun M, Dong F (2008). Sliding mode nonlinear friction compensation control of gun control servo system of tank. *Fire Control and Command Control*.

[9] Yuan D, Ma XJ, Li LY (2011). Adaptive compensation control method of nonlinearity in tank gun control system based on equivalent disturbance. *Fire Control and Command Control*.

[10] Gao Q, Chen J, Wang L, Xu S, Hou Y (2013). Multiobjective optimization design of a fractional order PID controller for a gun control system. *The Scientific World Journal*.

[11] Podlubny I (1999). Fractional-order systems and PI *λ*D *μ*-controllers. *IEEE Transactions on Automatic Control*.

[12] Zhou X, Zhu Z, Zhao S, Luo D A novel hybrid control strategy for trajectory tracking of fast tool servo.

[13] Calderón AJ, Vinagre BM, Feliu V (2006). Fractional order control strategies for power electronic buck converters. *Signal Processing*.

[14] Zhou X, Zhu Z, Zhao S, Lin J, Dou J (2011). An improved adaptive feedforward cancellation for trajectory tracking of fast tool servo based on fractional calculus. *Procedia Engineering*.

[15] Erenturk K (2013). Fractional order PI*λ*D*μ* and active disturbance rejection control of nonlinear two mass drive system. *IEEE Transactions on Industrial Electronics*.

[16] Luo Y, Chen YQ (2009). Fractional order [proportional derivative] controller for a class of fractional order systems. *Automatica*.

[17] Li H, Luo Y, Chen YQ (2010). A fractional order proportional and derivative (FOPD) motion controller: tuning rule and experiments. *IEEE Transactions on Control Systems Technology*.

[18] Luo Y, Chen YQ (2012). Stabilizing and robust fractional order PI controller synthesis for first order plus time delay systems. *Automatica*.

[19] Luo Y, Chen YQ, Pi Y (2011). Experimental study of fractional order proportional derivative controller synthesis for fractional order systems. *Mechatronics*.

[20] Shim H, Jo NH (2009). An almost necessary and sufficient condition for robust stability of closed-loop systems with disturbance observer. *Automatica*.

[21] Kim KS, Rew KH (2013). Reduced order disturbance observer for discrete-time linear systems. *Automatica*.

[22] Choi Y, Yang K, Chung WK, Kim HR, Suh IH (2003). On the robustness and performance of disturbance observers for second-order systems. *IEEE Transactions on Automatic Control*.

